# Communicating Physical Activity with Adolescents: What works? A scoping review protocol

**DOI:** 10.12688/hrbopenres.13594.1

**Published:** 2022-08-25

**Authors:** Caera Grady, Kwok Ng, Elaine Murtagh, Catherine Woods

**Affiliations:** 1 The Physical Activity for Health Research Cluster, Health Research Institute, Physical Education and Sports Sciences Department, University of Limerick, Limerick, Co. Limerick, V94 T9PX, Ireland; 2 School of Educational Sciences and Psychology, University of Eastern Finland, Joensuu, Kuopio, FI-70029, Finland; 3 Faculty of Education, University of Turku, Rauma, Finland, FI-26100, Finland

**Keywords:** Effective communication, Physical Activity Messaging, youth, post-primary

## Abstract

*Background:* Worldwide, adolescents are not meeting the minimum recommended physical activity (PA) guidelines to achieve health benefits. Awareness of the guidelines among adolescents is low, only 3.6% can report them correctly. The school is an ideal PA promotion setting, no other institute has the same reach or influence on the adolescent population. There is a need for an effective communication strategy for PA messages for adolescents. The purpose of this review is to explore how, when, who, where and what i.e. the content, context and mode of delivery of PA messages to communicate with adolescents to improve their awareness and understanding of PA that will potentially lead to an increase in PA levels. *Methods:* A scoping review was selected as the most appropriate methodology due to the broad nature of the research question. The PCC mnemonic (Population, concept, context), recommended by the Joanna Briggs Institute, was used to develop the search strategy and research question. This review will follow the scoping review framework developed by Arksey and O’Malley (2005) which was later updated by Levac *et al* (2010) to ensure the methods are systematic. It will also follow the PRISMA extension for scoping reviews checklist. Sources include databases (CINAHL, Education Source, Scopus, PubMED), grey literature from the World Health Organisation, Global Index Medicus and the reference lists of extracted articles will be checked from the year 1995 onwards. *Results: *A PRISMA flow diagram will demonstrate the final articles included and results will be presented and summarised as recurring themes. The results will be discussed in relation to existing literature and future implications for research, policy and practice. *Conclusion: *This will be the first review to explore the PA messaging context among adolescents and the findings will help inform a strategy for communicating PA to adolescents.

## Introduction

The health benefits of engaging in regular physical activity (PA) are widely documented ^1^. However, over 80% of adolescents (11–17yrs) worldwide do not meet the PA guidelines to achieve these health benefits ^2^. The complex physical inactivity challenge has no single solution. The Global Action Plan for Physical Activity recommends a systems approach and multi-level stakeholder support through community wide interventions and national strategies to tackle the problem (low levels of PA) which involves creating connections, interactions and feedback between system architects ^3, 4^. Adolescence is an important life stage where PA patterns track into adulthood, researchers have called for an effective communication strategy for disseminating the message to ensure that young people are aware of and understand the PA recommendations ^5– 7^. A research study conducted in Portugal during the 2011–2012 academic year showed that less than 4% of adolescents were able to correctly identify the PA guidelines for children and adolescents with no statistically significant differences between gender or socio-economic status ^6^.

The school is an ideal setting for PA promotion albeit a unique, complex and adaptive sub-system thus, a systems approach is essential to increase population levels of PA among children and adolescents ^8, 9^. There are a wide range of professionals outside of the health sector with a potential role in promoting PA to adolescents, such as teachers within the school setting ^10^. A large proportion of children and adolescents’ waking day revolves around school; no other institution has the same reach or influence over this population ^5^. The International Society for Physical Activity and Health recommends whole-of-school programmes as one of the eight best investments for PA promotion among children and adolescents, this is a multi-component approach that aims to promote PA to all members of the school community ^11^. This can include physical education programs, active classrooms, after school PA opportunities, activities during break times and promoting active travel ^11^. Some examples of whole-of-school programmes include the Active School Flag (Ireland), Finnish Schools on the Move (Finland), Sigue la Huella (Spain) and the Comprehensive Schools Physical Activity Promotion (USA) working towards this approach ^12– 15^. Finally, curriculum subjects such as physical education and wellbeing are important opportunities for the promotion of PA to adolescents within the school setting. In order to support the promotion of PA in schools the needs within the school setting must be understood. One such solution is to identify acceptable and effective communication methods and tools that align with the strategic priorities and routine work within the school ^10^.

PA messaging has been described as ‘the overall process of creating and delivering PA messages’ and has potential to improve population PA levels ^16^. Previous reviews have provided recommendations for improving the communication of PA messages to the general public, underserved communities, parents and children with disabilities ^10, 16– 21^. Some approaches used when communicating messages in the aforementioned studies include formative research, social marketing principals, message tailoring and message framing.

i. *Formative research* is the act of gathering data that can be used for the development and implementation of programmes ^22^. It can help determine the appropriate message content, context and delivery methods suitable for the target population ^16^.
ii. *Social marketing* uses commercial marketing ideas and strategies to promote voluntary behavioural change; it involves the exchange of a product (i.e. PA) for a cost (i.e. time or energy) in a manner that is attractive to an individual ^23, 24^. It uses affective and emotional responses to make a product more appealing ^16^. The VERB campaign is an example where this approach was successful in promoting PA as enjoyable and fun to adolescents ^25^. After four years of the campaign 75% or more tweens (9–13yrs) were aware of and understood the VERB messages and in the final year, 28% had unprompted recall of VERB ^26^.
iii. *Message tailoring* or targeting presents information that is suitable to the individual characteristics of the message recipient ^18^. Tailoring messages has been proven to increase message salience and the impact of the message on the behaviour change ^27, 28^. Although message tailoring should be considered a promising practice with potential to enhance the effectiveness of messages accompanying the PA guidelines, it is not essential ^18^.
iv. *PA message framing*, originating from Prospect Theory, suggests that individuals will respond differently to factually equivalent messages depending on whether they are worded to highlight benefits or consequences ^16, 18, 29^. Gain framing is generally preferred over loss-framing messages when promoting PA potentially due to its inclusion of information targeting the psychological determinants of PA ^18^.

Each of these approaches contribute to understanding the needs within the target group, the development and delivery of acceptable and effective communication methods and tools to support PA promotion. There is a need to explore the literature to determine which methods are suitable to ensure effective communication with adolescents in order to increase PA knowledge, awareness, attitudes and levels.

### Rationale for this scoping review

A scoping review addresses broader topics where many different study designs might be applicable and is unlikely to address specific research questions nor assess the quality of included studies ^30^. In contrast, a systematic review would typically focus on a well-defined question, can identify study designs, with the aim to provide answers to the questions from a narrow range of quality assessed studies. Due to the broad nature of the research topic, a scoping review was identified as the most appropriate methodological approach. It will allow us to explore the breadth or depth of the literature, map and summarise the evidence, inform future research and identify and address knowledge gaps ^31^.

Preliminary searches were conducted by the primary author, on communicating PA messages to adolescents across various databases like CINAHL, Scopus, PubMed. The searches revealed no reviews conducted that focused solely on PA messages for the adolescent population in general. Reviews that targeted PA messaging with other population groups meeting our eligibility criteria e.g. children with physical disabilities, were identified and will be included in the review ^16, 21^. Little evidence identifying the most effective methods of communicating PA messages to adolescents i.e. content, context and mode of delivery was found. Thus, this review will help determine the extent of the literature among adolescents and clarify the key concepts of communicating messages to adolescents.

### Scoping review aims

This research aims to explore existing messaging techniques or strategies in PA promotion interventions for adolescents, their implementation and effectiveness in leading to a positive PA behaviour change.

### Objectives

The specific objectives are to:

1. Identify and map the literature on how messages have previously been communicated to adolescents in relation to PA
2. Clarify the methods and approaches used in previous studies that involved communicating messages to adolescents.
3. Discuss the evaluation of the outcomes measured in the articles identified within the scoping review.

## Methods

### Protocol

This scoping review will follow the guidelines and stages set out by Levac, Colquhoun ^32^ who built upon the methodological framework set out by Arksey and O'Malley ^30^. This updated framework has six essential stages: i) identifying the research question, ii) identifying relevant studies, iii) study selection, iv) charting the data, v) collating, summarising and reporting results and vi) consultation with knowledge users. The preferred reporting items for systematic reviews and meta-analysis have developed an extension for scoping reviews (PRISMA-ScR) 22-item checklist will also be utilised in this study ^31^.

***Step 1: Identifying the research question***

The “PCC” mnemonic (population, concept and context) was used to develop a clear title and research question ^33^. In order to capture the most appropriate body of literature we iteratively searched the literature and revised search terms which resulted with the main concept of communicating messages and principal contextual settings for which the concept will be explored: PA promotion programmes or interventions and the target population as adolescents. Finally, our outcome of interest is to determine what, who, when, where and how PA messages should be communicated to adolescents.

The following research questions were identified:

*1. What (content), how (mode of delivery/communication) and who/when/where (context) to deliver PA messages to adolescents?*

*2. What methodologies are used to determine the effectiveness of the messages for adolescents? (i.e. an instrument)*

*3. Should a mass media or multi-media communication campaign be included in whole-school PA programmes? (i.e. are they effective?)*

***Step 2: Identifying relevant studies***

For comprehensiveness and rigor several databases: Scopus, EBSCO ( CINAHL, Education source), PubMED will be searched. We will also include grey literature such as conference abstracts, theses and reports from the World Health Organisation Global Index Medicus. Table 1 in the *Extended data* gives an overview of the predetermined eligibility criteria i.e., inclusion/exclusion criteria.

***Step 3: Study selection***

Using an iterative process we identified and agreed upon a search strategy as a team in collaboration with the faculty librarian in the School of Education and Health Sciences at the University of Limerick (Extended data, Appendix 1) ^32^. Firstly, a preliminary search of the literature was conducted on CINAHL searching article titles, abstracts and full texts to guide the development of our second search strategy. Second, we are including the identified keywords and subject headings in the search strategy across all databases being used. Finally, will look at the reference lists from articles selected for the review i.e. snowballing. The librarian provided suggestions and verifications regarding the appropriate syntax and the adaption of search strategies across databases.

Once the searches have been ran in all databases, the search results will be downloaded to EndNote where duplicates will be removed and then uploaded to Rayyan review software where if necessary further duplicates will be removed ^34^. The title/abstract screening process will be piloted by double screening 10% of retrieved articles (10% by CG and 10% by either KN or EM) and inter-rater reliability calculated for title and abstract screening using kappa statistic. Screening will begin once an agreement rate of 75% or greater by CG and either EM/KN as per pre-determined eligibility criteria. If it is unclear whether or not to include an article based on the first stage of the reviewing process, the study will be included for full-text review to ensure it is not being excluded without full consideration. The same process will be repeated when screening full text articles with reviewers from the research team. The reviewers will meet at multiple stages throughout the reviewing process to discuss any discrepancies that may have emerged. Any discrepancies will be discussed until a consensus is achieved, arbitrated as necessary by a third reviewer.

***Step 4: Preliminary charting elements and associated question***

An Excel table (see *Extended data,* Table 2) will be used to extract and chart the data systematically. The rows will represent the different articles and the columns will provide key details addressed in the article such as, the context, content, mode of delivery, method of measurement, implementation and effectiveness of PA messages. This information is important to note for the next stage of the review process. The piloting process will take place with two or more researchers charting the data independently from the first 5–10 studies and meet to determine whether their approach to data extraction is consistent with the research question and purpose. This stage is an iterative process as new data are presented in the examination stages, leading to continual charting updates.

***Step 5: Collating summarising and reporting the results***

This stage has three clear steps:

   i)     Collating and summarising the results

Following data extraction and charting we will provide a narrative synthesis of the included studies, descriptively summarising the data that has been charted. We will not critically appraise the data; we will look to aggregate the findings of the included studies allowing us to summarise and identify recurring themes. These themes will be reported qualitatively and displayed in a way which answers the proposed research question and objectives.

   ii)     Reporting the results

Results will be reported using the PRISMA-ScR guidelines and a PRISMA flow diagram will be included to show all studies selected or excluded at each stage ^35^. Findings will be organised into categories such as aims, methodological design, key findings and gaps in the literature but also by categories that specifically highlight theoretical and operational linkages such as context, conceptual and operational features and measurements used.

   iii)     Research implications for future research, practice and policy

By understanding how PA messages are delivered in what context and by whom will help inform the development of a PA messaging framework for adolescents. The broader implications of the findings for policy, practice and research will be highlighted.

***Step 6: Consultation with knowledge users***

Levac *et al*. suggested this stage should be essential, after Arksey and O’Malley deemed it as optional, as it adds to the methodological rigour of a study ^30, 32^. Since this review is a part of a study on effective PA communication for adolescents, we will utilise an existing partnership between researchers, knowledge users and post-primary schools. This collaboration initially began in 2018 where formative participatory research took place with the University of Limerick researchers, and the students, teachers, parents and sports coaches in Irish post-primary schools.

The format for consulting with stakeholders will follow a participatory approach in the style of a workshop with focus group discussions. It will include opportunities for learning and action using tools for presenting the results of the scoping review and discussion between stakeholders about any potential modifications regarding how the literature defines, measures and operationalises effective communication with adolescents. The purpose of this consultation is to share preliminary findings with stakeholders, validate the findings i.e. ensure they have practical relevance and to inform a draft effective PA communication for adolescent’s toolkit. A further series of consultation phases will take place with additional stakeholders as part of a follow-up study i.e. a modified delphi study.

## Discussion

The school is an ideal PA promoting setting, whole-of-school programmes and Physical Education curricula play a large role in the way PA is communicated to adolescents ^11, 36^. Previous literature highlights the importance and greater effectiveness of portraying PA as fun and enjoyable to children and adolescents rather than stating the health-related benefits of PA ^16^. This review aims to map and synthesis the available literature in relation to the specific content of PA messages that is deemed acceptable by adolescents, the delivery of the message i.e. the most appropriate personnel and the method in which it is communicated e.g. multi-media campaign and the environment in which the messages are communicated e.g. whole school PA programmes. The findings from this review will guide the direction of future research with adolescents to identify an effective communication strategy for PA.

## Ethics and dissemination

Ethical approval was not required or obtained for this scoping review. The scoping review findings will be disseminated in a peer-reviewed journal and the findings will be presented at appropriate academic conferences and to project partners to inform the design of the effective PA communication for adolescent’s toolkit. As highlighted above in *Step 6: Consultation with knowledge users* we will share the findings with an array of stakeholders who will have the opportunity to discuss potential modifications for action.

## Data availability

### Underlying data

No data are associated with this article.

### Extended data

Open Science Framework: Communicating Physical Activity with Adolescents: What works? A scoping review protocol. https://doi.org/10.17605/OSF.IO/BCNS6 ^35^

Extended data (available on the OSF registration):

- Table 1. eligibility criteria (pre-determined inclusion/exclusion criteria)
- Table 2. Data charting elements (variables to be extracted from the included studies)
- Appendix 1. Scopus search strategy (complete search strategy in Scopus database)
- Appendix 2. PRISMA-P 2015 checklist

Data are available under the terms of the Creative Commons Attribution 4.0 International license (CC-BY 4.0).

## References

[1] Bull FC Al-Ansari SS Biddle S : World Health Organization 2020 guidelines on physical activity and sedentary behaviour. *Br J Sports Med.* 2020;54(24):1451–62. 10.1136/bjsports-2020-102955 33239350 PMC7719906

[2] Guthold R Stevens GA Riley LM : Global trends in insufficient physical activity among adolescents: a pooled analysis of 298 population-based surveys with 1· 6 million participants. *Lancet Child Adolesc Health.* 2020;4(1):23–35. 10.1016/S2352-4642(19)30323-2 31761562 PMC6919336

[3] World Health Organisation: Global action plan on physical activity (GAPPA) 2018–2030: more active people for a healthier world. Geneva: World Health Organization;2018. Reference Source

[4] Murphy JJ Barry N Bauman A : Getting Ireland Active'- Application of a systems approach to increasing physical activity in Ireland using the GAPPA framework. *J Phys Act Health.* 2021;18(11):1427–1436. 10.1123/jpah.2020-0864 34583322

[5] Dobbins M Husson H DeCorby K : School-based physical activity programs for promoting physical activity and fitness in children and adolescents aged 6 to 18. *Cochrane Database Syst Rev.* 2013;2013(2):CD007651. 10.1002/14651858.CD007651.pub2 23450577 PMC7197501

[6] Marques A Martins J Sarmento H : Do students know the physical activity recommendations for health promotion? *J Phys Act Health.* 2015;12(2):253–6. 10.1123/jpah.2013-0228 24769866

[7] Hayes G Dowd KP MacDonncha C : Tracking of Physical Activity and Sedentary Behavior From Adolescence to Young Adulthood: A Systematic Literature Review. *J Adolesc Health.* 2019;65(4):446–54. 10.1016/j.jadohealth.2019.03.013 31248803

[8] Daly-Smith A Quarmby T Archbold VSJ : Using a multi-stakeholder experience-based design process to co-develop the Creating Active Schools Framework. *Int J Behav Nutr Phys Act.* 2020;17(1):13. 10.1186/s12966-020-0917-z 32028968 PMC7006100

[9] van Sluijs EMF Ekelund U Crochemore-Silva I : Physical activity behaviours in adolescence: current evidence and opportunities for intervention. *Lancet.* 2021;398(10298):429–42. 10.1016/S0140-6736(21)01259-9 34302767 PMC7612669

[10] Milton K Bauman AE Faulkner G : Maximising the impact of global and national physical activity guidelines: the critical role of communication strategies. *Bri J Sports Med.* 2020;54(24):1463. 10.1136/bjsports-2020-102324 33239351 PMC7719904

[11] Milton K Cavill N Chalkley A : Eight Investments That Work for Physical Activity. *J Phys Act Health.* 2021;18(6):625–30. 10.1123/jpah.2021-0112 33984836

[12] Ng KW McHale F Cotter K : Feasibility Study of the Secondary Level Active School Flag Programme: Study Protocol. *J Funct Morphol Kinesiol.* 2019;4(1):16. 10.3390/jfmk4010016 33467331 PMC7739289

[13] Pardo BM Clemente JAJ González LG : Development of the 'Sigue la Huella' physical activity intervention for adolescents in Huesca, Spain. *Health Promot Int.* 2019;34(3):519–531. 10.1093/heapro/day005 29529200

[14] Haapala HL Hirvensalo MH Kulmala J : Changes in physical activity and sedentary time in the Finnish Schools on the Move program: a quasi-experimental study. *Scand J Med Sci Sports.* 2017;27(11):1442–53. 10.1111/sms.12790 27781314

[15] America SoHaPES: Comprehensive School Physical Activity Program (CSPAP): Centre of Disease Control (CDC) & SHAPE America. Reference Source

[16] Williamson C Baker G Mutrie N : Get the message? A scoping review of physical activity messaging. *Int J Behav Nutr Phys Act.* 2020;17(1):51. 10.1186/s12966-020-00954-3 32295613 PMC7160981

[17] Nobles J Thomas C Banks Gross Z : "Let's Talk about Physical Activity": Understanding the Preferences of Under-Served Communities when Messaging Physical Activity Guidelines to the Public. *Int J Environ Res Public Health.* 2020;17(8):2782. 10.3390/ijerph17082782 32316591 PMC7215851

[18] Latimer AE Brawley LR Bassett RL : A systematic review of three approaches for constructing physical activity messages: What messages work and what improvements are needed? *Int J Behav Nutr Phys Act.* 2010;7:36. 10.1186/1479-5868-7-36 20459779 PMC2885311

[19] Latimer-Cheung AE Rhodes RE Kho ME : Evidence-informed recommendations for constructing and disseminating messages supplementing the new Canadian Physical Activity Guidelines. *BMC Public Health.* 2013;13:419. 10.1186/1471-2458-13-419 23634998 PMC3654879

[20] Bergeron CD Tanner AH Friedman D : Physical Activity Communication: A Scoping Review of the Literature. *Health Promot Pract.* 2019;20(3):344–353. 10.1177/1524839919834272 30832516

[21] Larocca V Arbour-Nicitopoulos K Latimer-Cheung A : Physical Activity Messages for Youth with Disabilities: An Evaluation of Attitudes, Intentions, and Preferences. *Health Commun.* 2020;35(8):974–983. 10.1080/10410236.2019.1598746 30961368

[22] Gittelsohn J Steckler A Johnson CC : Formative research in school and community-based health programs and studies: "state of the art" and the TAAG approach. *Health Educ Behav.* 2006;33(1):25–39. 10.1177/1090198105282412 16397157 PMC2475675

[23] Andreasen AR : Social Marketing: Its Definition and Domain. *J Pub Pol Market.* 1994;13(1):108–114. 10.1177/074391569401300109

[24] Brawley LR Latimer AE : Physical activity guides for Canadians: messaging strategies, realistic expectations for change, and evaluation. *Can J Public Health.* 2007;98 Suppl 2:S170–84. 18213947

[25] Huhman M Kelly RP Edgar T : Social Marketing as a Framework for Youth Physical Activity Initiatives: a 10-Year Retrospective on the Legacy of CDC's VERB Campaign. *Curr Obes Rep.* 2017;6(2):101–107. 10.1007/s13679-017-0252-0 28421471

[26] Huhman ME Potter LD Duke JC : Evaluation of a National Physical Activity Intervention for Children: VERB™ Campaign, 2002–2004. *Am J Preven Med.* 2007;32(1):38–43. 10.1016/j.amepre.2006.08.030 17218189

[27] Kreuter MW Wray RJ : Tailored and targeted health communication: strategies for enhancing information relevance. *Am J Health Behav.* 2003;27 Suppl 3:S227–32. 10.5993/ajhb.27.1.s3.6 14672383

[28] Noar SM Benac CN Harris MS : Does tailoring matter? Meta-analytic review of tailored print health behavior change interventions. *Psychol Bull.* 2007;133(4):673–93. 10.1037/0033-2909.133.4.673 17592961

[29] Tversky A Kahneman D : The framing of decisions and the psychology of choice. *Science.* 1981;211(4481):453–8. 10.1126/science.7455683 7455683

[30] Arksey H O'Malley L : Scoping Studies: Towards a Methodological Framework. *Int J Society Res Meth: Theory Pract.* 2005;8(1):19–32. 10.1080/1364557032000119616

[31] Tricco AC Lillie E Zarin W : PRISMA Extension for Scoping Reviews (PRISMA-ScR): Checklist and Explanation. *Ann Intern Med.* 2018;169(7):467–73. 10.7326/M18-0850 30178033

[32] Levac D Colquhoun H O'Brien KK : Scoping studies: advancing the methodology. *Implement Sci.* 2010;5:69. 10.1186/1748-5908-5-69 20854677 PMC2954944

[33] Peters MDJ Marnie C Tricco AC : Updated methodological guidance for the conduct of scoping reviews. *JBI Evid Synth.* 2020;18(10):2119–2126. 10.11124/JBIES-20-00167 33038124

[34] Ouzzani M Hammady H Fedorowicz Z : Rayyan — a web and mobile app for systematic reviews. *Syst Rev.* 2016;5(1):210. 10.1186/s13643-016-0384-4 27919275 PMC5139140

[35] Grady CL Ng K Murtagh E : Communicating Physical Activity with Adolescents: What Works? A Scoping Review Protocol. OSF. [Data].2022. 10.17605/OSF.IO/BCNS6 PMC9672528 36452513

[36] Centers for Disease Control and Prevention (CDC): School health guidelines to promote healthy eating and physical activity. *MMWR Recomm Rep.* 2011;60(RR–5):1–76. 21918496

