# Supplementary material for: Communicating Physical Activity with Adolescents: What works? A scoping review protocol
Source: HRB Open Res. 2022 Aug 25;5:59. [Version 1] doi: 10.12688/hrbopenres.13594.1 (PMC9672528; doi:10.12688/hrbopenres.13594.1)
Supplement: Peer review history

# Peer review: Reviewer response for version 1

**DOI:** 10.21956/hrbopenres.14851.r33093

**Published:** 2022-11-17

**Authors:** Leanne C. Doherty

**Affiliations:** 1 The Centre for Exercise Medicine, Physical Activity and Health, Sport and Exercise Sciences Research Institute, Ulster University, Coleraine, UK

***Is the rationale for, and objectives of, the study clearly described?***

 Yes. The authors adequately outlined the problem of physical inactivity in this cohort of adolescents and the need to identify effective techniques to communicate with them, to promote physical activity, and in turn impact behavior. This research is very relevant given the rate of obesity and physical inactivity globally. 

***Is the study design appropriate for the research question?***

 Yes. The authors clearly explained the difference between a scoping review and a systematic review and clearly justified their choice of undertaking a scoping review.

***Are sufficient details of the methods provided to allow replication by others?***

 Yes. The authors clearly outline how they will adhere to the methodological framework for conducting scoping reviews. I was easily able to access the supplementary files regarding eligibility criteria, data charting elements, and Scopus search strategy. I think the reporting plan is very appropriate and given the data will be organised by categories and linked to the review objectives, the results will be well-structured and easy to follow. This will aid the reader when relating the findings to the stated objectives.

**Additional comments**

 I have one comment regarding the inclusion criteria. Your population/sample will include adolescents in post-primary schools, yet the review is not limited to the school setting. Would it, therefore, be more beneficial to replace "in post-primary schools" with the appropriate age range? Some adolescents leave the post-primary setting at 15/16 years of age to move into other Further Education facilities.

Is the study design appropriate for the research question?

Yes

Is the rationale for, and objectives of, the study clearly described?

Yes

Are sufficient details of the methods provided to allow replication by others?

Yes

Are the datasets clearly presented in a useable and accessible format?

Not applicable

Reviewer Expertise:

Human Nutrition, Physical activity and Health research.

I confirm that I have read this submission and believe that I have an appropriate level of expertise to confirm that it is of an acceptable scientific standard.

# Peer review: Reviewer response for version 1

**DOI:** 10.21956/hrbopenres.14851.r32742

**Published:** 2022-08-26

**Authors:** Matthew Mclaughlin

**Affiliations:** 1 Telethon Kids Institute, The University of Western Australia, Callaghan, NSW, Australia

**Is the rationale for, and objectives of, the study clearly described?**

 Yes, the study outlines the need for a review in this population of messaging/communicating physical activity. I particularly enjoyed the succinct writing style, kudos to the authors for packing a lot of information into such a short paper.

**Is the study design appropriate for the research question?**

 Yes, a sufficient rationale for a scoping review is provided (e.g. compared with a systematic review).

**Are sufficient details of the methods provided to allow replication by others?**

 Yes, the tables will be particularly useful for replication purposes. Although the clear use of scoping review methodologies and checklists is also to be commended.

**Are the datasets clearly presented in a useable and accessible format**?

 Yes, this is a protocol.

**Major comments**

 Overall this is an excellent protocol and I am confident it will be a useful contribution to the literature. I only have one question/idea/suggestion, which may have been considered already, but I'd be keen to see the authors thoughts: Can the authors provide more detail on the grey literature they plan to search and what strategy they will follow? I ask because the description in Table 1 implies that government reports, websites, books etc. will not be searched. I imagined there would be some publicly available reports from government and NGO's that may have conducted campaigns? Anyway, just a thought! Not a dealbreaker for this protocol. Just a possible way to boost the number of included articles.

Is the study design appropriate for the research question?

Yes

Is the rationale for, and objectives of, the study clearly described?

Yes

Are sufficient details of the methods provided to allow replication by others?

Yes

Are the datasets clearly presented in a useable and accessible format?

Yes

Reviewer Expertise:

Physical activity, young people, reviews, interventions, scale-up.

I confirm that I have read this submission and believe that I have an appropriate level of expertise to confirm that it is of an acceptable scientific standard.
